# Early Childhood Caries among a Bedouin community residing in the eastern outskirts of Jerusalem

**DOI:** 10.1186/1471-2458-7-167

**Published:** 2007-07-24

**Authors:** Alon Livny, Rula Assali, Harold D Sgan-Cohen

**Affiliations:** 1 Affiliation of all authors: Department of Community Dentistry, Hebrew University- Hadassah School of Dental Medicine, Jerusalem, Israel

## Abstract

**Background:**

ECC is commonly prevalent among underprivileged populations. The Jahalin Bedouin are a severely deprived, previously nomadic tribe, dwelling on the eastern outskirts of Jerusalem. The aim of this study was to assess ECC prevalence and potentially associated variables.

**Methods:**

102 children aged 12–36 months were visually examined for caries, mothers' anterior dentition was visually subjectively appraised, demographic and health behavior data were collected by interview.

**Results:**

Among children, 17.6% demonstrated ECC, among mothers, 37.3% revealed "fairly bad" anterior teeth. Among children drinking bottles there was about twice the level of ECC (20.3%) than those breast-fed (13.2%). ECC was found only among children aged more than one year (p < 0.001); more prevalent ECC (55.6%) was found among large (10–13 children) families than among smaller families (1–5 children: 13.5%, 6–9 children: 15.6%) (p = 0.009); ECC was more prevalent among children of less educated mothers (p = 0.037); ECC was more prevalent among mothers with "fairly poor" anterior dentition (p = 0.04). Oral hygiene practices were poor.

**Conclusion:**

ECC levels in this community were not very high but neither low. This changing population might be on the verge of a wider dental disease "epidemic". Public health efforts clearly need to be invested towards the oral health and general welfare of this community.

## Background

Early Childhood Caries (ECC) is a serious public health problem in both developing and industrialized countries. It continues to affect babies and preschool children worldwide. ECC prevalence varies from population to population; however, children of disadvantaged subpopulations, regardless of race, ethnicity or culture, have been found to be most vulnerable [1]. In these populations, prenatal and perinatal malnutrition are often the causes of enamel hypoplasia, reduced salivary secretion and low buffering capacity [2-6]. Additionally, oral hygiene is usually poor, exposure to fluoride is probably insufficient, and general psychosocial stress is common [7,8]. These variables may impede the natural resistance to the disease cycle of bacterial invasion, demineralization, and dental caries [2].

Untreated ECC can lead to harsh consequences such as abscesses, pain, malocclusions and lasting psychosocial impediments [9,10]. Since the level of cooperative behavior of babies and preschool children is less than ideal, the current standard of care for treatment of ECC often necessitates general anesthesia, with its potential complications and costs [11]. Clinical outcomes for treatment of ECC are often poor, and several studies have reported that 23% – 57% of children treated under general anesthesia require further treatment for new carious lesions within 6–24 months [12-15].

The definition of ECC is sometimes ambiguous, and over the years different case definitions have been suggested and applied. This has often caused difficulties in comparing epidemiologic data from different studies [16]. In 1999, a US government sponsored (NIDCR) workshop addressed this issue and suggested defined ECC as the presence of one or more carious tooth surfaces in any primary tooth [17], which was adopted in the present study.

Bedouin are Arab nomadic communities, found throughout most of the desert belt extending from the Atlantic coast of the Sahara to the eastern coast of the Arabian Desert. Bedouin have traditionally avoided localized agricultural work, but policies of the various Middle Eastern states since the 20th century have obligated many of them into a more sedentary life.

The present study focused on the Bedouin tribe of Al-Jahalin, which includes about 2,200 inhabitants residing in an area east of Jerusalem. During the last five decades, the Jahalin, originally dwelling in the "Negev" desert of southern Israel, were gradually coerced to move from their original wider surroundings and environment, mainly due to political and military reasons. Since 1995 they are all concentrated on one barren hill in the Judean desert, east of Jerusalem, a region currently of contested "ownership" within the Israeli-Palestinian conflict.

The majority of this community lives in self-built shanty huts or freight containers supplied by the Israeli government. Almost none of them continue to herd sheep, and most earn their living by working in adjacent towns [18-20].

This is an extremely disadvantaged community, lacking basic facilities such as proper water supply, electricity and sewerage infrastructure. Basic education and health services are minimal or absent. In general, the majority of these people are of low education level [19].

Breast-feeding is very common among Bedouin mothers. Usually, after delivery the breast-feeding continues until the next pregnancy [21]. The Jahalin diet mainly consists of homemade "Pitah" bread, rice, beans, lentils and vegetables. Those who rear goats, also consume milk and its homemade products. The Jahalin, as all Bedouins, are very fond of sweetened tea, which is also their main item in welcoming guests or strangers, along with fresh goat milk. Some of the more well-off families can afford to buy candies, chocolate, and soft drinks for their children. On site there is one shop, which mainly sells refreshments and soft drinks, brought from the nearby town. Previous research on Bedouins, especially in rural areas, observed very low caries prevalence, attributed to the traditional diet which is of abrasive nature and low in refined carbohydrates [22,23].

The objectives of this pilot study were to assess the prevalence of ECC among 12–36-month-old children among the Al-Jahalin Bedouins, and associations with potentially related factors

## Methods

This was a cross-sectional survey, among children aged 12–36 months, of the Al- Jahalin Bedouin tribe in the area on the eastern outskirts of Jerusalem. As no estimates of the total number of children within this age range were available before the study, attempts were made to visit all families and include all children of the age group. Parents were previously informed about the study aims and all gave their informed consent. Permission was received from the appropriate authorities.

Data were collected by means of visual clinical examinations, preceded by short interviews with the mothers. All oral examinations were carried out within the homes by one examiner (RA). The examination was conducted in a visual non-tactile manner commonly referred to as "lift the lip" technique [24]. Mouth mirrors were used for indirect vision of lingual areas of the teeth, and penlight was used for lighting. Infants were examined on their mothers' lap, by means of the "Knee-to-knee" technique [25]. Gauze pads were used to clean and dry teeth surfaces prior to examination. Carious lesions, by tooth, were recorded according to the WHO (dmft) criteria [26]. Enamel hyperplasia or white spot lesions were not recorded as caries. ECC was defined according to the NIDCR guidelines [17].

Prior to the oral examination, mothers were interviewed for the following information: family demographics, breast-feeding and bottle-feeding practices, types of food, level of dental care and hygiene practices (of both mothers and children). In addition, the appearance of the mother's upper anterior teeth was assessed subjectively by the examiner, and served as a proxy measurement of mother's attitude towards dental health. The examiner looked at the mother's anterior teeth and recorded their appearance. Clearly visible anterior caries or missing teeth were operationally defined as "fairly bad". Present intact anterior dentition was defined as "fairly good".

A pilot survey was conducted among 10 children, in order to pretest the method of examination, the data collection forms, and to train and calibrate the examiner. This pilot stage was conducted among a same age group of Arab children, in a "Mother and Child Health" Center in Jerusalem, which was considered as an analogous population. Only one of the ten children examined presented caries, in three teeth. Full agreement was reached between the study examiner and the experienced dental epidemiologist (AL).

Following data collection and coding, statistical analysis was applied, employing the Statistical Package for the Social Sciences (SPSS), version 12.0 for Windows. Percentage distributions of sample characteristics and associations between different factors and caries presence were computed, applying statistical tests of Chi-square and Pearson's correlation. Variables which had revealed significant univariate associations were included in the multivariate analysis of logistic regression model. Statistical significance level for all tests was chosen as p < 0.05.

## Results

### Study population

The study population comprised of 102 children, 56 males and 46 females (54.9%, 49.1%), aged 12–36 months. This represented the total population (total number of target population was unavailable and therefore "compliance" level could not be ascertained). Of these, 48 children (46.5%) were aged 12–24 months, and 54 (53.5%) were aged 25–36 months. Most of the children were part of large households: 74 families (75%) had 1–5 children, 19 (19%) had 6–9 children and nine (9%) had ten or more children. Two thirds of the children (68, 66.7%) lived in shanty huts, 26 (25.3%) lived in concrete houses, two (2%) in tents, and six (6%) in a combination of the above. Only two mothers had completed more than 12 years of education, 24 (23.5%) completed 10–12 years, 50(49%) completed 1–9 years, and 26 (25.5%) had no formal education at all. Similar education levels were reported for the fathers, except that ten (9.8%) of the latter had no formal education.

### Diet

Breast-feeding was the norm in this community and this practice included 96 of the study children (94%). At the time of the interviews, 23.5% of the study children were currently being breast-fed. Almost all of them (99%) were fed both at day and night, mostly (90%) "On demand". More than half of the study children (64, 62.7%) were bottle-fed. The majority of these (59/64, 92%) were also breast-fed. Bottles contained milk exclusively for 48% of the children, or in combination with herb tea (35%) or canned juice (17%).

Mothers were asked to name five types of food that their child ate regularly, and 94 (92.2%) reported a home-prepared "non-cariogenic" (no added "extrinsic" sugar) diet, while eight children (7.8%) reportedly ate commercially bought processed "cariogenic" food. All children drank local tap water. Water samples from the site were analysed and we found a less than optimal fluoride level of 0.3 ppm.

### Oral hygiene practices and dental care

The majority of the children (95, 93%) did not have their teeth regularly cleaned. Out of the seven children whose mothers reported teeth cleaning, six used a toothbrush and fluoridated toothpaste, and one used only water, five of the seven children had their teeth cleaned twice a day, morning and evening. Most of the mothers (97, 95%) reported cleaning their own teeth regularly. Of these – 53% cleaned teeth daily, 18.6% did so three times a week, and 28.4% less than three times a week. Among mothers who cleaned their teeth, 83 (85.6%) used a toothbrush and toothpaste, 12 (12.4%) used "Siwak" in addition to tooth brushing (The "Siwak" or "Miswak" are tree twigs which are widely used among Moslems as a device to clean their teeth), one used a mouthwash in addition to tooth brushing, and one mother only used Siwak.

Assuming the subjective appearance of the upper anterior teeth as an initial impression of the mothers' dental condition, 64 (62.7%) mothers revealed a "fairly good" condition, and 38 (37.3%) a "fairly bad" condition. Mothers were also asked about dental treatments over the past two years, and 37% reported that they had not been to the dentist, 32.4% had been to the dentist three times or more, 22% once, and 9% twice. They had all visited private dentists (there were no public services available), and the cause for all visits was dental pain. Most of the treatments performed were exclusively extractions (71.8%).

### Caries prevalence

**Figure 1:**
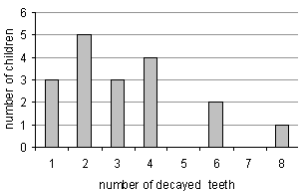
Distribution of decayed teeth among the affected children.

Eighteen of the 102 children (17.6%) demonstrated ECC, and 84(82.4%) were caries-free. Caries was more prevalent in males (21%) than in females (13%), but this difference did not reach statistical significance. All children with caries were in the older age group of 25–36 months. In all, there were 58 decayed teeth; none of these had ever been treated. The maximum caries level per child (dmft) was eight teeth. The mean dmft among the whole group was 0.57 teeth (58/102). Among the 18 children who presented caries – the mean was 3.2 teeth (58/18). The SiC (Significant Caries Index = the average caries level among one third of the population with the most caries [27]) in this population was calculated to be 1.7 (58/34). Figure 1 presents the distribution of the carious teeth among the affected children.

### Associations

Caries levels differed by the various reported feeding practices and higher levels of caries were seen among children whose mothers reported bottle feeding, but these differences were not statistically significant (Table 1). A significant statistical association was found between type of housing and bottle use: families living in concrete houses used bottles for feeding infants less than families living in shanty huts or tents (*p *= 0.001).

**Table 1:** Associations between caries and feeding practices

Feeding practices	Caries	Caries free	Total	statistics
Bottle use:				
Yes	13 (20.3%)	51 (79.7%)	64 (100%)	
No	5 (13.2%)	33 (86.8%)	38 (100%)	n.s.*
				
Breast feeding:				
Yes	16 (16.5%)	81 (83.5%)	97 (100.0%)	
No	2 (40.0%)	3 (60.0%)	5 (100.0%)	n.s.
				
Breast feeding Only	5 (13.2%)	33 (86.8%)	38 (100.0%)	
Bottle only	2 (40.0%)	3 (60.0%)	5 (100.0%)	
Both	11 (18.6%)	48 (81.4%)	59 (100.0%)	n.s.

Total:	18 (17.6%)	84 (82.4%)	102 (100%)	

* not statistically significant

Caries was significantly associated, according to univariate analysis (Table 2), with four factors: children's age, family size, mothers' education level and mothers' dental appearance. These factors were therefore considered in the multiple regression model. Only one mother among the caries group completed more than 10 years of education, therefore this variable was not included in the regression model. The age factor was also excluded, since among the entire younger group was caries free.

**Table 2:** Associations between caries and independent factors

	Caries	Caries free	statistics
Age:			
12–24 months	0	47 (100%)	χ^2 ^test:
25–36 months	18 (33.3%)	37 (66.7%)	p < 0.001
			
Family size:			
1–5 children	10 (13.5%)	64 (86.5%)	Linear by linear ass:
6–9 children	3 (15.8%)	16 (84.2%)	p= 0.009
10–13 children	5 (55.6%)	4 (44.4%)	
			
Mother's Educational level:			
0–9 years	17 (22.4%)	59 (77.6%)	Fisher's Exact Test:
10 or more years	1 (3.8%)	25 (96.2%)	p= 0.037
			
Mother's teeth appearance:			
Fairly good	6 (9.4%)	58 (90.6%)	χ^2 ^test:
Fairly poor	12 (31.6%)	26 (68.4%)	p= 0.04

Table 3 displays the results of the multiple regression analysis. Children from families of more than five children were about ten times more likely to have caries than children from smaller families, children of mothers whose front teeth looked "fairly good", were four times more likely to be caries free. These differences, however, did not reach statistical significance.

**Table 3:** Multiple regression analysis of association between caries and two independent variables

	Presence of caries			
	
	N	OR	Sig.	95% CI
Family size (number of children):				
1–5	10	1		
6–9	3	10.89	n.s.*	0.48 – 248.23
10–13	5	9.48	n.s.	0.62 – 144.82
				
mother's teeth appearance:				
Fairly good	6	1		
Fairly poor	12	0.25	n.s.	0.02 – 3.27

* not statistically significant

## Discussion

This study examined Early Childhood Caries among children aged 12–36 months in the community of the Bedouin tribe of Al-Jahalin, settled in the eastern outskirts of Jerusalem. Regarding the extreme poverty and minimal living standards it had been assumed that dental disease could be high. On the other hand, recognizing that caries is a disease of modern "westernization", it had also been considered that levels might be lower. According to the results, ECC prevalence was 18%. This level is higher than formerly reported in several European countries [28,29], but lower than among several native North-American communities [1,30,31]. A recent study, conducted among a representative sample of 596 Jewish children aged 2.5–3 years in Jerusalem, found an ECC prevalence of 15.3%, which is not very much lower than results of the present study [32]. The findings of this study are not dissimilar to results of research conducted among Bedouin children in other regions [33,34]. Dental caries was higher than found in earlier Israeli studies (from the 1960's and 1980's) conducted among Bedouin groups, where caries experience had been described as "very slight" [21,22].

Recent studies from European countries, as well as from North America, reveal an increasing pattern of ECC levels, significantly associated with race and ethnicity [9,35-38].

The prevalence of caries among Al-Jahalin Bedouins was not low and could be associated with their changing daily life. The traditional nomadic style has changed; they have access to commercial cariogenic foods and beverages; they do not rely exclusively on home-made food as in the past; they have adopted "modern" practices such as bottle feeding [21,23].

Prolonged breast feeding has been reported to be associated with severe caries by a few authors [39]. We found differences in caries levels in relation to type of feeding, which were not statistically significant. The reason for this may be the small population size, particularly the small group of children who were fed exclusively by bottle, in addition to the small number of children observed to be affected by caries. However, several studies have suggested that the previously presumed association between caries and type of feeding was not significant [40,41].

Only 20.3% of children who were fed baby bottle milk on demand (alone or in combination with breast feeding) had caries. This finding supports the observation that sleeping with baby bottle milk or other sugary drinks does not necessarily cause caries [42].

The practice of tooth brushing with fluoridated toothpaste was very low among the children in this study. The vast majority of the examined children (93%) did not have their teeth cleaned at all. During the interviews, most mothers demonstrated lack of knowledge about the importance of cleaning infants' teeth or teaching them to brush teeth at an early age. This finding is unacceptable from a public health point of view. Studies from the UK and Sweden reported tooth brushing was as high as 90–95% among 18-month-old children [43,44]. Tooth brushing by parents or caregivers has the potential of removing dental plaque more effectively, optimally saturating the oral environment with fluoride, and thereby decreasing the risk of caries among their children [44,45].

The low level of tooth brushing may also be due to limited access to community tap water. These inhabitants have to walk with buckets or containers to fill water from a remote single water pipe. Water limitation most likely would impair daily tooth brushing, by both mothers and infants.

The present survey examined the associations between demographic or social factors and caries prevalence. Only four variables reached levels of statistical significance: age of the children, mothers' education level, family size, and the subjective appearance of mothers' teeth. Caries lesions were found only among children older than one year of age. ECC was more prevalent among children of mothers with a lower education level. Studies have shown an increased risk of ECC associated with low economic income, low social class and mothers' education [45,46].

A significant association was demonstrated between children's caries level and mother's teeth appearance (as subjectively assessed by the examiner). Children of mothers with "fairly good" appearance of teeth had less caries than children of mothers with "fairly poor" appearance. Dental appearance could be interpreted as the mother's awareness of self-image and dental care, both for herself and for her child. Furthermore, mothers with "fairly poor" appearance are assumed to harbor higher levels of pathological bacteria with the potential of contaminating their children. Several studies have found associations between mothers' and their children's dental status [6,47].

Family size was strongly associated with caries level. The number of children has been commonly recognized as an indicator of socioeconomic level, but also as a contributor to health behavior of mothers. The average number of children in this study population was 4.6 and the maximum was 13. It could be assumed that in a large family it is more difficult for the parents to provide optimal individual health care according to each child's needs, including healthy feeding and oral hygiene practices.

Multiple regression results showed that mother's "fairly poor" dental appearance and large family size were associated with higher caries presence (four and ten times more, respectively), but without reaching statistical significance. This was presumed to be due to the relatively low number of caries cases in each group. The low number of subjects (N = 102) could certainly have influenced the statistical significance of findings. This could not have been prevented, as the study was conducted among the whole target population and not a sample. Pooling together additional similar tribes could have produced larger numbers, but it must be considered that each Bedouin community is different in its characteristics.

The nature of this Bedouin community, which is facing cultural and sociological transitions, can be compared with other indigenous ethnic groups, experiencing transitions accompanied by oral health deterioration [9,35,48]. This is an issue of global importance which should not be ignored.

One of the inescapable major problems of this community is that, besides being culturally and materially disadvantaged, it also suffers from severe political and organizational uncertainty, within the context of the current Israeli-Palestinian conflict. Despite the destitution of this community, all children have the basic right to health, welfare and quality of life. This is the basic public health goal and premise [49].

## Conclusion

This is a pioneer survey which examined ECC among preschool Bedouin children. This changing population might be on the verge of a more highly prevalent epidemic of dental disease. The levels of ECC among the Al-Jahalin Bedouin children, the lack of optimally fluoridated water, the scarceness of adequate oral hygiene practices, and the apparent shift towards bottle feeding, all indicate that community efforts should be attempted and evaluated. These could include: water fluoridation, oral health education of mothers, promotion of using fluoridated dentifrice, dietary counseling, self-examination for early signs of ECC, and other previously tested methods which are potentially simple, cheap, effective and appropriately practical for this community. The general improvement of this community's quality of life is an essential basic goal.

Although countless obstacles exist, results of this study may be helpful in encouraging and persuading the related authorities about the needs of this community. Interceptive prevention programs should be planned and the present baseline data would be employed for future continued follow-up and evaluative research.

## Competing interests

The author(s) declare that they have no competing interests.

## Authors' contributions

AL was the principal investigator; RA conducted the field examinations; HSC was the chief supervisor, participated in the design of the study and performed the data analysis. All authors read and approved the final manuscript.

## Pre-publication history

The pre-publication history for this paper can be accessed here:


